# Association of Adiponectin and rs1501299 of the *ADIPOQ* Gene with Prediabetes in Jordan

**DOI:** 10.3390/biom8040117

**Published:** 2018-10-22

**Authors:** Mahmoud A. Alfaqih, Faheem Al-Mughales, Othman Al-Shboul, Mohammad Al Qudah, Yousef S. Khader, Muhammad Al-Jarrah

**Affiliations:** 1 Department of Physiology and Biochemistry, School of Medicine, Jordan University of Science and Technology, Irbid 22110, Jordan; fahmughales@gmail.com (F.A.-M.); oashboul@just.edu.jo (O.A.-S.); 2 Department of Family Medicine and Public Health, School of Medicine, Jordan University of Science and Technology, Irbid 22110, Jordan; Maalqudah7@just.edu.jo (M.A.Q.); yskhader@just.edu.jo (Y.S.K.); 3 Department of Rehabilitation Sciences, School of Applied Medical Sciences, Jordan University of Science and Technology, Irbid 22110, Jordan; jarrahm@just.edu.jo

**Keywords:** diabetes mellitus, prediabetes, insulin resistance, adiponectin, *ADIPOQ*, single nucleotide polymorphisms, rs1501299

## Abstract

Type 2 diabetes mellitus (T2DM) is a worldwide health problem caused by resistance to insulin action. This chronic debilitating diseaseis preceded by a stage, known as prediabetes, in which a healthy lifestyle can delay the disease. The discovery of biochemical changes in prediabetes is important to identify individuals at risk of developing T2DM and in explaining disease pathogenesis. Adiponectin is secreted by fat cells and is linked with insulin resistance. Adiponectin levels are dysregulated in prediabetic subjects. This relationship had not been tested in Jordan. We recruited 130 subjects with prediabetes and 130 control subjects. We measured serum levels of adiponectin and genotyped subjects for three single nucleotide polymorphisms (SNPs) in the *ADIPOQ* gene; rs266729, rs1501299 and rs2241766. In multivariate analysis, we found that serum adiponectin lowers the risk of prediabetes (*p* = 0.002; odds ratio (OR), 0.764; 95% confidence interval (CI), 0.646–0.905). The rs1501299 SNP of the *ADIPOQ* gene was associated with prediabetes in our population (*p* = 0.041). Specifically, in multivariate analysis, the GT genotype of rs1501299 increased the risk of prediabetes (*p* = 0.010; OR, 2.350; 95% CI, 1.231–4.486) as well as the TT genotype (*p* = 0.006; OR, 4.774; 95% CI, 1.551–14.693). Our findings indicate that serum adiponectin and SNPs in the *ADIPOQ* gene are associated with prediabetes in Jordan.

## 1. Introduction

The most recent statistics that describe the prevalence of diabetes mellitus (DM) clearly show that this chronic disease is becoming a worldwide health problem of epidemic proportions [1]. In 2015 it was estimated that 415 million people are living with DM worldwide [2]. The same study reported that 75% of DM patients live in countries of low-to-middle income, with the highest prevalence rates recorded in the Oceania and the Middle East and North Africa (MENA) regions [2]. An increased life expectancy together with a sedentary lifestyle and a Western-based diet are thought to be responsible for a DM epidemic in Jordan; a developing country in the MENA region [3]. In Jordan, the prevalence of DM has increased from 13% to 17% over a 10-year period (1994–2014) [4]. These figures clearly emphasize the need for better strategies not only to treat DM, but also to prevent it by cost-effective measures and public educational programs.

The majority of diabetic patients (around 90% of DM cases) are of type 2 DM (T2DM) caused by the resistance to insulin action in peripheral tissues, and DM mostly affects obese adults [5]. Prediabetes is a stage that precedes T2DM. In prediabetes, resistance to insulin action is compensated by higher levels of insulin in the serum. [6]. Prediabetes is a stage where active intervention programs consisting of a healthy diet and exercise can delay or stop the progression of the disease into “frank” diabetes [6]. The discovery of early biochemical changes that accompany this stage of the disease is important to identify individuals who are at risk of developing DM and who would benefit from the above intervention programs [6]. The above biochemical markers may also be of utility in (a) monitoring the progression of the disease, and (b) explaining disease pathogenesis. Building on the above discussion, biochemical markers that accompany insulin resistance, a hallmark of prediabetes [7], provide a fertile soil for the identification of disease biomarkers of prediabetes. 

Adiponectin belongs to a class of peptide hormones secreted by fat cells collectively known as adipocytokines [8]. Adiponectin, the most abundant adipocytokine, increases insulin sensitivity on its target tissues; a feature that might explain the lower levels of adiponectin commonly observed in obese individuals [8] and in individuals suffering from T2DM [9]. Not surprisingly, lower levels of serum adiponectin were reported in prediabetic individuals of several populations [10]. This association, however, had not been explored in Jordan despite the size of the DM problem and its impact on the country’s health sector. It is worth mentioning that serum adiponectin levels and presumably the risk of developing insulin resistance and consequent disease sequelae may also be influenced by single nucleotide polymorphisms (SNPs) in *ADIPOQ*, the gene that codes for the adiponectin protein [11]. Indeed, our group was the first to report that the rs1501299 SNP of the *ADIPOQ* gene is associated with polycystic ovarian syndrome (PCOS) [11], a disease strongly linked with insulin resistance [12]. Not all PCOS women have prediabetes. In this study, we hypothesized that serum adiponectin levels are lower in prediabetes subjects, regardless of their gender, compared to nondiabetic controls of a Jordanian population, and that SNPs in the *ADIPOQ* gene may modify the levels of serum adiponectin in prediabetic subjects and consequently the risk of developing prediabetes.

## 2. Materials and Methods

### 2.1. Study Design, Subject Description and Collection of Blood and Serum Samples

This was a prospective case-control study in design. The cases were not matched with the controls. The project was approved by the appropriate Institutional Review Boards, project identification code (21 March 2017) affiliated with the Jordan University of Science and Technology (JUST) (Irbid, Jordan). Date of approval of the Institutional Review Board (IRB) was on the twentieth of February of the year 2017. Participation in the study required an informed consent. Recruitment of prediabetic and control subjects took place at the Family Medicine clinics of King Abdullah University Hospital (KAUH); a teaching hospital under the affiliation of JUST considered the biggest medical facility in Northern Jordan. Recruitment took place between April 2017 and February 2018.

Subjects were first interviewed by a clinical research coordinator who verbally explained the objectives of the study. Demographic data, anthropometric measurements (height, weight and waist circumference (WC)) and relevant medical history were collected into a structured data collection sheet. During the subjects’ next visit to the clinic, a blood sample (5 mL) was withdrawn into an Ethylene-Diamine-Tetra-Acetic acid (EDTA) tube (AFCO, Amman, Jordan), and another sample (5 mL) was withdrawn into a plain tube with gel clot activator (AFCO, Amman, Jordan) following an overnight fast of 14-h duration. The sample in the EDTA tube was stored at 4 °C and was later used for DNA extraction. Serum was separated from the blood sample collected in the plain tube following centrifugation at 4000 *g* for 7 min. Collected serum was used to measure fasting glucose, total cholesterol and triglyceride levels. The remaining serum was divided into 1.5 mL Eppendorf tubes and stored at −80 °C. Serum in Eppendorf tubes was later used for adiponectin measurements. Subjects that had a fasting serum glucose level between 100–125 mg/dL were asked to repeat the test at their next visit to the clinic and were considered to have prediabetes if fasting glucose levels remained between 100–125 mg/dL. Subjects that had fasting serum glucose levels below 100 mg/dL were considered to be free of diabetes and were enrolled in the control arm of the study. Subjects with DM as indicated by a fasting serum glucose level of 126 mg/dL or above at two separate visits, or as revealed by their medical history, were excluded from both arms of the study. Subjects with Cushing’s syndrome, syndromes of severe insulin resistance, androgen secreting neoplasms, thyroid dysfunction, congenital adrenal hyperplasia, or hyperprolactinemia were also excluded from both arms of the study. The following formula, Body Mass Index (BMI) = weight (kg)/[height (m)]^2^, was used for BMI calculation.

A total of 260 subjects enrolled in this study; 130 subjects had prediabetes and 130 were free from prediabetes and served as controls. We used the following formula (SD^2^(Z_α/2_ + Z_β_)^2^/d^2^) to calculate the minimum required sample size to detect a difference between cases and controls at a confidence level of 95%. The standard deviation (SD) and the effect size (d) were estimated to be 2.61 and 0.66 based on previous local and regional studies [13,14,15]. In the above formula, α was assumed to be at 5% and β at 20%. Using the above formula, the minimum sample size was 123 subjects per group.

The power to detect the association between each allele and prediabetes was calculated using the Genetic Association Study (GAS) Power Calculator (http://csg.sph.umich.edu/abecasis/gas_power_calculator/). We assumed that the prevalence of prediabetes is 20% in Jordan [3]. In this study, the frequencies of alleles that were associated with higher risk of prediabetes were 0.35 (the T allele for rs1501299) and 0.79 (the A allele for rs266729). Using the selected sample size of 130 cases and 130 controls at a corrected level of significance of 0.025 (this was corrected for two SNPs because rs2241766 allele frequency deviated from Hardy–Weinberg Equilibrium (HWE)), the power to detect odds ratio of 2.0 between each allele and prediabetes exceeded 80%.

### 2.2. Biochemical Measurements

The collected serum was used to measure the levels of glucose, total cholesterol, triglycerides and adiponectin. Measurement of serum glucose, total cholesterol and triglycerides was performed on a Roche automated clinical analyzer system (Roche Diagnostics, Mannheim, Germany). Serum adiponectin levels were estimated using an enzyme-linked immunosorbent assay (ELISA) purchased from R&D Systems (Minneapolis, MN, USA) as described in Alfaqih et al. [11]. Measurements were performed after an 8000-fold dilution of the serum samples. Dilutions were prepared in phosphate-buffered saline containing 0.1% bovine serum albumin. The absorbance was measured using an ELx800 microplate reader (BioTek Instruments, Winooski, VT, USA) at a wavelength of 450 nm.

### 2.3. DNA Extraction and Genotyping

Blood in EDTA tubes was used to purify genomic DNA using QIAamp DNA Blood Mini Kit purchased from Qiagen (Hilden, Germany) according to the instructions of the manufacturer. Following DNA extraction, the final DNA concentration was measured spectrophotometrically using an ND-2000 Nanodrop (Thermo Scientific, Waltham, MA, USA). Genotyping of all three SNPs of *ADIPOQ* (rs266729, rs1501299, rs2241766) was performed using polymerase chain reaction–restriction fragment length polymorphism (PCR–RFLP). Details of the PCR–RFLP assay including the location of the SNPs on the *ADIPOQ* gene, the sequence of the PCR primers, the size of the PCR amplicon, the restriction enzyme used for genotyping each SNP and the size of the products following restriction enzyme digestion can be found in Table 1 and in Alfaqih et al. [11]. The undigested PCR amplicons and the DNA fragments following restriction enzyme digestion were electrophoresed on a 3% agarose gel containing ethidium bromide. Ultraviolet light was used to visualize the products.

### 2.4. Statistical Analysis

The Statistical Package for Social Studies (SPSS) software (version 22, IBM, Armonk, NY) was used to conduct all statistical analyses. A Student’s *t*-test was used to examine if a statistically significant difference is present in serum glucose, total cholesterol, triglycerides, adiponectin, age, gender, BMI or WC between prediabetes and control subjects. Pearson’s chi squared test was used to examine if rs266729, rs1501299 or rs2241766 conformed to HWE. Pearson’s chi squared was also used to test if an association existed between different genotypes or allele categories of rs266729, rs1501299 or rs2241766 with prediabetes.

Multivariate logistic regression analysis was used to determine if serum adiponectin and rs1501299 remained associated with prediabetes following adjustment with age, gender, BMI, WC, serum cholesterol, serum triglycerides and rs266729. The multicollinearity was tested by using multiple linear regression for the set of independent factors excluding the original response and allowing one of the factors as response. The analysis showed that the problem is minimal and this was evident by the fact that when we added or removed each factor from the model, the regression coefficients did change significantly. A *p*-value of less than 0.05 was considered statistically significant.

## 3. Results

### 3.1. Subject Characteristics and Biochemical Profile

Significant differences existed in gender distribution between prediabetic and control subjects (*p* = 0.0064) (Table 2); the majority of prediabetic subjects were males (58%), while the majority of control subjects were females (59%). Prediabetic subjects were significantly younger than controls (*p* = 0.0482) and had a significantly higher BMI (*p* < 0.0001) and WC (*p* < 0.0001). The biochemical profile showed that prediabetic subjects had significantly higher levels of fasting serum glucose (*p* < 0.0001) but significantly lower levels of serum adiponectin (*p* < 0.0001). Serum cholesterol and triglycerides were not significantly different between prediabetic and control subjects.

### 3.2. Association of Adiponectin and ADIPOQ Gene Variants with Prediabetes

We next wanted to determine if *ADIPOQ* gene variants were associated with prediabetes. We genotyped prediabetic and control subjects for three SNPs in the *ADIPOQ* gene (rs266729, rs1501299, rs2241766) previously tested for their association with PCOS in Jordan [11]. Like prediabetes, insulin resistance also plays a role in PCOS pathogenesis [16]. The rs2241766 SNP significantly deviated from HWE (*p* < 0.0001), as shown in Table 3. Thus, rs2241766 was excluded from further analysis. Genotype frequencies of rs266729 and rs1501299 are shown in Table 4, while the allele frequencies are shown in Table 5. Genotype call rates and allele call rates of all three SNPs were 100%. Significant differences existed in genotype frequencies (*p* = 0.041) and allele frequencies (*p* = 0.0168) of rs1501299 between control and prediabetic subjects.

It was previously shown that serum adiponectin levels could be affected by age [17], gender [17,18] and BMI [19]. Control and prediabetic subjects in our study population were not matched for age, gender, BMI or WC. Therefore, we wanted to test if adiponectin and *ADIPOQ* gene variants remain associated with prediabetes following the adjustment for the above variables. To achieve this goal, we used multivariate regression analysis with age, gender, BMI, WC, total cholesterol, triglycerides and adiponectin in the model. We also included rs266729 and rs1501299 *ADIPOQ* SNPs. In this analysis, we showed that serum adiponectin was associated with a lower risk of prediabetes (*p* = 0.002; OR, 0.764; 95% CI, 0.646–0.905) (Table 6). Furthermore, our results indicated that the GT genotype of rs1501299 increased the risk of prediabetes relative to the GG genotype (*p* = 0.010; OR, 2.350; 95% CI, 1.231–4.486). The TT genotype also increased the risk of prediabetes (*p* = 0.006; OR, 4.774; 95% CI, 1.551–14.693) (Table 6).

## 4. Discussion

The findings of this investigation add to a growing body of evidence that a tentative relationship exists between lower serum adiponectin levels and prediabetes [10,20]. Our results highlight a serum marker that may aid in discovering individuals at a higher risk of developing T2DM in the future or in monitoring disease progression. Moreover, our finding that an SNP in *ADIPOQ*, rs1501299, was associated with prediabetes risk in our univariate and multivariate models further highlights the tentative role that serum adiponectin plays in the pathogenesis of prediabetes, and provides a potential genetic marker of prediabetes in Jordan.

Dysregulation in serum adiponectin in prediabetes may be involved in disease pathogenesis or may be a result of insulin resistance observed in prediabetic individuals. However, it remains possible that lifestyle modification and/or pharmacological modalities that increase serum adiponectin levels reduce the risk of prediabetes or even delay/prevent the progression of prediabetes into T2DM. In this context, it is of significance to note that agonists of peroxisome proliferator-activated receptor-gamma (PPARɣ) (example: thiazolidinediones) are reported to increase the serum levels of adiponectin [21]. The clinical utility of administering PPARɣ agonists to prediabetic individuals as a means to prevent/delay the progression of the disease to T2DM is still too early and requires well-designed clinical trials across multiple ethnicities. Lifestyle modifications that modify the secretory profile of adipose tissue and increase the levels of secreted adiponectin may be a viable alternative to PPARɣ agonists, especially considering that such an intervention has a better safety profile. Indeed, the use of PPARɣ agonists is associated with an increased risk of pulmonary edema and congestive cardiac failure; two potentially lethal side effects [22,23]. Alternatively, a lower dose of PPARɣ agonists may be used in combination with lifestyle modification to reduce the risk of the above side effects of this class of medications. 

In addition to our finding that the rs1501299 SNP of *ADIPOQ* was associated with prediabetes in Jordan, we previously reported that rs1501299 was also associated with PCOS in Jordanian women [11]. Moreover, Khabour et al. demonstrated that rs1501299 was associated with longevity in Jordanian men [24]. A common feature of all of the above conditions is that they all could be affected/modulated by insulin resistance [12,25]. This observation requires confirmation using epidemiology-based approaches such as meta-analysis, but is certainly worthy of further investigation, especially if the effect of rs1501299 turns out to be ethnicity specific. Such a finding will also emphasize studies that attempt to decipher the exact mechanism by which rs1501299 modulates energy metabolism and/or insulin resistance. Notably, several studies from multiple populations found that rs1501299 is associated with lower levels of serum adiponectin [26,27,28]. Thus, the association that we found between rs1501299 and prediabetes could be explained by the result of the association between rs1501299 and hypoadiponectineamia. The mechanism by which rs1501299 affects serum adiponectin levels remains to be elucidated. The rs1501299 SNP is located in the first intron. Although not part of the promoter, rs1501299 could be located on an enhancer sequence. Enhancer sequences could be located in introns and could thus modulate gene expression [29]. Moreover, rs1501299 could give rise to alternatively spliced mRNA [30] or affect mRNA stability [31]. It is more informative to investigate the above tentative mechanism using animal- and organ/cell-based models.

Serum adiponectin levels appear to have a strong genetic component. Indeed, estimates of heritability of adiponectin levels range between 30–50% [32]. Considering the strong association between serum adiponectin levels and prediabetes, and our finding that rs1501299 is associated with prediabetes in our population and several other Asian populations, it could be hypothesized that rs1501299 could serve as a genetic marker of prediabetes; this, however, requires further validation.

In addition to rs1501299, we tested the association of one other SNP in *ADIPOQ* (rs266729) with prediabetes. The genotype and allele frequencies of rs266729 were not different between prediabetic subjects and healthy controls, indicating a lack of association. Several reasons could explain this observation; (i) rs266729 may not have a direct effect on adiponectin expression/activity in contrast to rs1501299, (ii) the effect of rs1501299 on prediabetes could be specifically modified by the presence of other SNPs in the vicinity of the same genomic area, and (iii) the effect of rs266729 may be small and would thus require a more highly powered study design to detect an association.

Notably, serum levels of adiponectin are not only affected by obesity but also are modulated by fat distribution (visceral vs subcutaneous fat) [33]. Visceral and subcutaneous fat cannot be distinguished using BMI and WC. [34]. Although our results showed that adiponectin remained associated with prediabetes independent of BMI and WC, differences in fat distribution between prediabetic and control subjects may still account for the differences we observed in serum adiponectin levels. Future studies should thus include measurements that reflect differences in fat distribution, such as waist-to-hip ratio [35]. Another limitation of our investigation is the small sample size.

## 5. Conclusions

In conclusion, our research group is the first to report differences in serum adiponectin levels between prediabetic and control subjects in Jordan. These differences were independent of age, gender, BMI and WC of the subjects. We are also the first to report that the rs1501299 SNP of *ADIPOQ* was associated with prediabetic independent of all of the above covariates. 

## Figures and Tables

**Table 1:** *ADIPOQ* SNP information.

SNP ID	Location and Base Change	Forward Primer Reverse Primer	PCR Product Size (bp)	Restriction Enzyme	RFLP Products (bp)
rs266729	Promoter * region (G/A)	ACTGTGGAGATGATATCTGG CATTTTGACAGCTACCTTGG	412	Hha1	GG: 170, 243 AG: 170, 243, 412 AA: 412
rs1501299	Intron 2 * (G/T)	TGACCAGGAAACCACGACTC CCATCTACACTCATCCTTGG	341	BsmI	GG: 229, 112 GT: 341, 229, 112 TT: 341
rs2241766	Exon 2 * (T/G)	AGTAGACTCTGCTGAGATGG ACATTCTTACCTGGATCTCC	333	BspH1	TT: 153, 180 TG: 333, 153, 180 GG: 333

* All SNP information was obtained from the NCBI dbSNP database, SNP: single nucleotide polymorphism, PCR: polymerase chain reaction, RFLP: restriction fragment length polymorphism.

**Table 2:** Baseline variables of study subjects.

Variable	Control (*n* = 130)	Prediabetes (*n* = 130)	*p*-Value ^1^
Gender (*n*) (%)			
Males	53 (41%)	75 (58%)	
Females	77 (59%)	55 (42%)	0.0064
Age (years)	53.24 ± 10.79 ^2^	50.82 ± 8.73	0.0482
BMI ^3^ (kg/m^2^)	29.62 ± 5.24	33.15 ± 6.26	<0.0001
WC ^4^ (cm)	101.70 ± 12.78	113.40 ± 13.46	<0.0001
Glucose (mg/dL)	86.81 ± 11.36	118.90 ± 19.78	<0.0001
Cholesterol (mg/dL)	183.90 ± 39.99	185.40 ± 42.32	0.7640
Triglycerides (mg/dL)	145.10 ± 114.46	153.40 ± 84.43	0.5080
Adiponectin (µg/mL)	4.66 ± 2.35	3.64 ± 1.49	<0.0001

^1^ The *p*-values were calculated by the Student’s *t*-test; ^2^ data are presented as the mean ± standard deviation; ^3^ BMI: body mass index; ^4^ WC: waist circumference.

**Table 3:** The *p*-value of the exact test of the Hardy–Weinberg equilibrium for rs266729, rs1501299 and rs2241766.

SNP ID	*p*-Value
rs266729	0.71
rs1501299	0.13
rs2241766	<0.0001

**Table 4:** Genotype frequencies of rs266729, rs1501299 and rs2241766 SNPs in control and prediabetic subjects.

SNP ID	Genotype	Control *n* (%)	Prediabetes *n* (%)	*p*-Value ^1^
rs266729	A/A	76 (58.5%)	88 (67.7%)	0.110
	A/G	45 (34.6%)	39 (30%)	
	G/G	9 (6.9%)	3 (2.3%)	
rs1501299	G/G	61 (46.9%)	43 (33.1%)	0.041
	G/T	60 (46.1%)	70 (53.9%)	
	T/T	9 (6.9%)	17 (13.1%)	

^1^*p*-values were calculated by the Pearson’s chi squared test.

**Table 5:** Allele frequencies of rs266729, rs1501299 and rs2241766 SNPs in control and prediabetic subjects.

SNP ID	Allele	Control *n* (%)	Prediabetes *n* (%)	*p*-Value ^1^
rs266729	A	197 (76%)	215 (83%)	0.0517
	G	63 (24%)	45 (17%)	
rs1501299	G	182 (70%)	156 (33.1%)	0.0168
	T	78 (30%)	104 (53.9%)	

^1^*p*-values were calculated by the Pearson’s chi squared test.

**Table 6:** Multivariate regression analysis of study subjects.

Variable	OR ^1^	95% CI ^2^	*p*-Value ^3^
Age (years)	0.961	0.931–0.992	0.013
WC (cm)	1.085	1.056–1.115	<0.001
Adiponectin (μg/mL)	0.764	0.646–0.905	0.002
rs1501299			
GG	1	-	
GT	2.350	1.231–4.486	0.010
TT	4.774	1.551–14.693	0.006
rs266729			
GG	1		
AG	1.417	0.248–8.104	0.695
AA	1.912	0.344–10.612	0.459

^1^ OR: odds ratio; ^2^ CI: confidence interval; ^3^ *p*-values were calculated by binomial logistic regression analysis; the results of only significantly associated variables are shown in the table.
